# The impact of experience quality on revisit intention: The chain mediation effect of tourist satisfaction and place attachment

**DOI:** 10.1097/MD.0000000000046477

**Published:** 2026-01-09

**Authors:** Yanqing Yan, Qiuxian Ye, Xiangfei Zhu, Jifeng Dong, Taiping Li

**Affiliations:** a Guangdong University of Science and Technology, Dongguan, Guangdong, China; b Zhuhai College of Science and Technology, Zhuhai, Guangdong, China.

**Keywords:** experience quality, outdoor camping tourism, place attachment, revisit intention, tourist satisfaction

## Abstract

Outdoor camping tourism, a nature-based leisure activity that promotes health, fosters social connections, and enhances environmental awareness, has experienced rapid growth in popularity. However, limited research has explored how tourists’ experience quality (EQ) shapes their behavioral intentions through psychological mechanisms. This study aimed to investigate the impact of EQ on revisit intention (RI) and to explore the mediating roles of tourist satisfaction (TS) and place attachment (PA) within the stimulus–organism–response theoretical framework. A structured questionnaire survey was conducted on a sample of 600 outdoor camping tourists in Guangdong Province, China. Partial least squares structural equation modeling was employed to test the hypothesized relationships and mediating effects. EQ significantly influenced RI (β = 0.236, *P* < .001) and indirectly affected RI through TS and PA. TS significantly enhanced PA (β = 0.473, *P* < .001), and the 2 variables jointly produced a chained mediating effect between EQ and RI (variance accounted for = 41.5%). Research confirms that high-quality experiences not only directly enhance tourists’ RIs but also cultivate satisfaction and PA, further enhancing loyalty to camping destinations. These findings enrich the application of stimulus–organism–response theory in tourism behavior studies, providing practical guidance for tourism managers to enhance EQ, strengthen emotional bonds, and promote the sustainable development of destinations.

## 1. Introduction

Guangdong Province is situated on the 3rd level of China’s terrain, characterized by generally low elevations. The complexity and diversity of its topography are influenced by a combination of factors, including crustal movements, magmatic activities, and changes in climate and sea level. Landforms primarily include mountains, hills, platforms, and plains, with mountains and hills comprising approximately 60% of the total land area of Guangdong Province, while platforms and plains account for about 40%. Guangdong Province has a subtropical monsoon climate, characterized by favorable temperatures throughout the year (average annual temperature of 21–23°C), with abundant but unevenly distributed precipitation. The climate is rainy in spring and summer, and dry in fall and winter. April and May, as well as September and October, are the best periods for camping, when the weather is mild and rainfall is low, making it suitable for outdoor activities. Guangdong boasts a variety of natural landscapes, including lakes, mountains, forests, and wetlands. Notable examples are Guangzhou’s Fenghuang Lake, renowned for its sea of bauhinia blossoms and sophisticated facilities, and Kowloon Lake, which attracts outdoor enthusiasts with its pristine ecology. Some campgrounds also offer complimentary activities, such as fishing and paddle boarding, which cater to diverse needs.^[1]^

Camping is a type of accommodation characterized by its simplicity and rusticity, attracting many people who appreciate the natural world.^[2]^ Camping tourism is a nature-based form of tourism that has become a popular leisure activity in recent years.^[3]^ Camping is categorized as a fairly healthy tourism activity and an economical and environmentally friendly leisure interest.^[4,5]^ Furthermore, camping tourism is described as a vacation activity. The shift from “exploring distant places” to “discovering nearby places” has facilitated the rapid growth of camping tourism.

From a tourist’s perspective, the primary benefits of camping tourism include improved physical and mental health, stress relief, and enhanced social connections.^[6,7]^ Participating in outdoor tourism activities can temporarily disconnect you from electronics, which can increase satisfaction and happiness while reducing stress. Camping with family or friends can also foster stronger relationships through shared experiences. On a societal level, camping tourism offers numerous benefits. It can be used as a tool to raise social awareness of sustainable tourism development, as campers can experience and appreciate the richness of natural resources through their camping trips,^[8]^ and people become more aware of their environmental impact. Raising awareness of ecosystems can significantly promote environmental protection.

Leisure researchers have begun to use the concept of PA to understand leisure travel behavior.^[9]^ Tourists who are satisfied with a destination are more likely to interact with the environment, develop a sense of attachment to that place, and return for another visit.^[10]^ Additionally, those who return to stay longer at a destination tend to have a stronger attachment to it.^[11]^ Camping tourism is an outdoor leisure activity, and PA has important implications when choosing a camping destination and will influence trip planning behavior. However, to date, few studies have considered the antecedents and outcomes of PA specifically within the context of camping tourism. In addition, there is not much discussion in the existing literature about the importance of experience quality (EQ) in tourist behavior.^[9,12]^ This study closes this gap in the literature by examining the relationship between EQ, TS, PA, and RI.

## 2. Literature review and hypothesis development

### 2.1. Theoretical backgrounds

Mehrabian and Russell proposed the stimulus–organism–response theory (SOR), which has been widely applied in psychology, consumer behavior, and tourism research.^[13]^ The core idea of this theory is that external environmental stimuli (S) evoke an individual’s internal cognitive and emotional states (O), which subsequently shape their behavioral responses (R). In the context of camping tourism, EQ can be viewed as the stimuli perceived by tourists during activities. Tourist satisfaction (TS) and place attachment (PA) represent internal organic states, while revisit intention (RI) constitutes the ultimate behavioral response. This framework provides a clear and concise logic for explaining how external experiences are internalized into psychological mechanisms and subsequently transformed into behavioral outcomes.

While interest in the application of SOR theory to tourist behavior has continued to expand, various research gaps remain. TS and PA have been studied as mediators between EQ and RI; however, only a limited number of studies have included both constructs within a chain mediation model.^[14,15]^ Moreover, while camping has emerged as one of the fastest-growing nature-based activities among tourists, research on outdoor camping remains limited. Addressing these gaps is valuable for advancing understanding of how tourist’ behavioral intentions are simultaneously influenced by both affective and cognitive mechanisms.

It is made possible to extend SOR theory to the field of tourism research by adding TS and PA into the chain mediation model. It is disclosed not only how TS and attachment are elicited by EQ, but also how internal procedures are transformed into higher RI. Furthermore, it provides a more comprehensive perspective upon the conversion of emotional–behavioral procedures in tourism while offering destination managers managerial implications to build sustainable tourist loyalty.

### 2.2. EQ and PA, TS, RI

Over the past few years, there has been an increasing volume of research on tourism EQ.^[16]^ In the tourism industry, scholars define EQ as the psychological and social reflection of the tourism experience. The ideas of EQ and service quality, while frequently conflated, are distinctly different. EQ derives not only from the various services enjoyed during tourism, but also from the interaction between tourists and the individuals, objects, and surroundings of the place. Thus, in terms of scope, EQ is broader than service quality. Despite the fact that research on the quality of tourists’ tourism experience has been quite extensive, there are still many potential influences that have yet to be identified. Satisfaction is a prerequisite for PA.^[17]^ Research supports the satisfaction and PA connection.^[18]^ However, research in the tourism industry generally recognizes that EQ has a positive impact on TS and RI^[19]^; however, TS has a positive impact on PA.

Therefore, this study proposes the following hypotheses:

H1. EQ will positively impact PA.

H2. EQ will positively impact TS.

H3. EQ quality will positively impact RI.

### 2.3. PA and RI

PA encompasses symbolism and expressing emotions. Hidalgo thinks that PA refers to the emotional bond persons form with a location.^[20]^ Gieryn states that PA is a person–place connection based on feelings (e.g., emotions), cognitions (e.g., knowledge and beliefs), and practices (e.g., actions and behaviors).^[21]^ Bingxi analyzed PA in waterparks, dividing factors into mental, functioning, and lifestyle attachments.^[22]^ Williams et al propose that location dependency and place identification form the basis of PA. Place dependence is a functional dependency that is based on how a tourist evaluates a particular place. Place identity refers to an individual’s emotional attachment to a location, which evolves through a process of self-adjustment. PA represents a multifaceted interplay between emotional expression and behavior.^[23]^ Hwang noted that the number of visits to a destination and the time spent there affect levels of attachment.^[24]^ High attachment may increase RI and ultimately increase loyalty to the destination.^[25]^ The deeper a tourist’s attachment to a particular location, the more frequently they visit that destination.^[26,27]^ Therefore, this study proposes the following hypotheses:

H4: PA will positively impact RI.

### 2.4. TS and PA, RI

TS and PA have completely attracted the interest of academic researchers and practitioners. According to several studies, TS may be a prerequisite for PA.^[28,29]^ According to Su et al, TS has an impact on both place identity and dependency.^[18]^ Furthermore, Hosany et al demonstrated that there is a beneficial effect of satisfaction among foreign tourists^[28]^, Zhang et al asserted that TS serves as a valuable indication in the context of festivals.^[29]^ For nature-based attractions to thrive in our competitive marketplace, customer satisfaction and PA strategies are imperative.^[30]^

The correlation between TS and behavioral intentions has been extensively studied.^[31]^ Fornell posits that higher TS with received services correlates with increased likelihood of favorable behavior towards the service provider and diminished probability of switching to an alternative provider.^[32]^ TS has been recognized as an important indicator of RI at the destination level^[33]^, which is reflected in many previous studies.^[31]^ If tourists have a positive experience at a place, they tend to return.^[34]^ Therefore, it is clear that the RI depends on TS^[35]^, which is related to PA.^[12]^ In this study, we propose the following hypotheses:

H5. TS will positively impact PA.

H6. TS will positively impact RI.

### 2.5. TS and PA, EQ, RI

Scholars have found that there is a correlation between EQ and TS and that when the service meets their expectations, it will result in favorable evaluations, which will lead to TS.^[36]^ These findings are supported by another study^[37]^, which states that the EQ scale consists of enjoyability, psychological peace, engagement, and identification. This study, utilizing research data from 3 primary tourism service sectors: accommodations, air travel, and attractions, demonstrates that EQ significantly impacts contentment, which subsequently enhances visitors’ RI.^[37]^ EQ, as an element of attraction, must stimulate tourists’ curiosity and allow them to engage in a personalized experience.

The desire to visit a place can be explained by intrinsic psychosocial factors such as PA.^[38]^ Psychologists have highlighted that PA serves as a significant motivator for individuals to return to and spend time in a location.^[39]^ Moulay and Ismail noted that the process of attachment formation affects the emergence of the behavior.^[40]^ Several scholars have validated this relationship, such as Prayag, who found a positive correlation between PA and RI in a study with tourists.^[41]^ His finding is supported by Loureiro study, which confirms that PA has a positive effect on RI.^[42]^ Additional research has investigated the indirect impacts of PA on behavioral intentions via TS^[43]^ and the mediating role of the relationship between TS and PA in behavioral intentions.^[44]^ However, these studies examined the relationship between PA, TS, and behavioral intentions in the context of national parks rather than camping tourism. More specifically, prior research has failed to test the mediating role of PA in the relationship between tourists’ RI.^[28]^ This study proposes the following hypotheses:

H7. PA plays a significant mediating role between EQ and RI.

H8. PA plays a significant mediating role between TS and RI.

H9. TS plays a significant mediating role between EQ and PA.

H10. TS plays a significant mediating role between EQ and RI.

H11. TS and PA have a chain mediating effect between EQ and RI.

**Figure 1.**
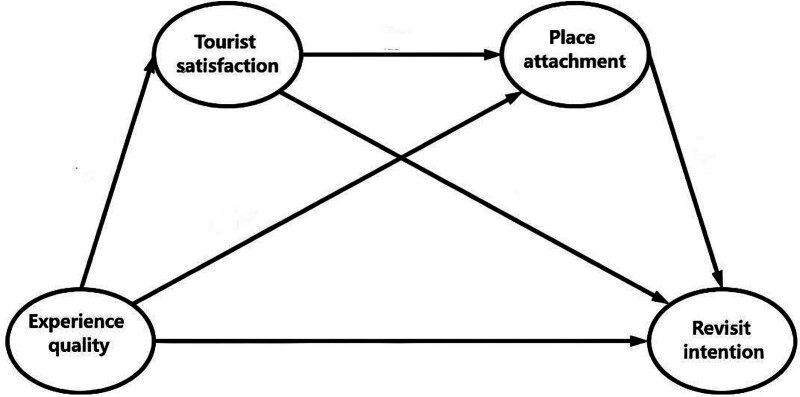
Conceptual research model. This figure illustrates the hypothesized relationships among experience quality, tourist satisfaction, place attachment, and revisit intention within the SOR framework. SOR = stimulus–organism–response theory.

Based on the theoretical structure and existing literature, this study formulates a series of hypotheses to investigate the effects of EQ, TS, PA, and RI of outdoor camping tourism in Guangdong Province, China. The constructed model is shown in Figure 1.

## 3. Research methodology

### 3.1. Sampling and data collection

We used convenience sampling in this survey. Convenience sampling saves money, effort, and time.^[45]^ The sample size was determined using the rule of 10 method, which has been employed in other investigations. This strategy requires a single item to receive 10 responses.^[46]^ We surveyed to gather data from outdoor camping enthusiasts in Guangdong Province. The questionnaire was created using Star Survey (www.wjx.cn), and the distribution of the link utilized multiple Chinese social media websites, including WeChat. We informed the participants that answering the questionnaire was voluntary. In addition, we ensured the confidentiality of the data by excluding their name, titles, telephone numbers, and email addresses from the questionnaire, and we promised the participants that their responses would solely be utilized for data analysis. Xia et al also collected relevant data in the tourism field through the Star Survey platform.^[47]^

### 3.2. Common method bias

We chose outdoor camping enthusiasts from different cities in Guangdong Province to minimize bias in the data collection process, which would increase the generalizability of the study.^[45]^ Moreover, we collected the data for the constructs at various time intervals (5 waves), a method that reduces bias.^[46]^ We track IP addresses and prohibit repeat submissions from the same applicant to avoid any bias.

### 3.3. Data analysis tools

This study uses IBM SPSS Statistics (Version 29.0; IBM Corp., Armonk) for descriptive statistics on the data. Additionally, we will use SmartPLS 4.1 software for the measurement model and structural evaluation during the data analysis process. This work applies partial least squares structural equation modeling. This survey selected this software for analysis because it has been successful in evaluating validity and reliability, and in confirming or rejecting hypotheses.^[48]^ In terms of analytical advantages, partial least squares can simultaneously estimate the path coefficients of the specified model and the loadings of individual items. Consequently, it enables researchers to circumvent biased and inconsistent parameter estimates^[49]^ and applies to more advanced and complex models.^[50]^

### 3.4. Measures

We used a 5-point Likert scale, where 1 represents “strongly disagree” and 5 represents “strongly agree.” EQ has been assessed using 5 items adapted from Parasuraman et al.^[51]^ In order to assess the TS, 4 items that were modified from Westbrook and Oliver^[52]^ were used. PA has been assessed using 4 items adapted from Vada et al.^[53]^ RI has been assessed using 3 items adapted from Moon and Han.^[54]^

### 3.5. Demographic characteristics of the sample

This research employed convenience sampling methods to gather data from persons possessing outdoor camping experience in Guangdong Province, China. A total of 600 valid replies were obtained via on-site and online methods. Specifically, 45 responses were obtained through direct face-to-face interaction with visitors at the outdoor camping locations. An additional 125 respondents were contacted in person at numerous outdoor camping locations, but chose to complete the survey online. The remaining 430 responses were collected through an online questionnaire with participants from social media platforms and online communities focused on outdoor camping tourism and nature tourism. The age distribution of the participants was diverse, with the largest groups being 26 to 30 years old (n = 172), 19 to 25 years old (n = 157), and 31 to 35 years old (n = 139). Notably, there were participants under the age of 18 (n = 86), while there were fewer in the 36 to 40 (n = 32) and 41 and over (n = 14) age groups. In terms of gender, the sample was more balanced, including 283 males and 317 females.

Participants were mainly from Guangdong Province, with the majority living in Guangzhou (324) and Dongguan (169). There were also a small number of participants from other cities, including Shenzhen (24), Shantou (30), Foshan (16), and Zhongshan (8). Zhuhai, Jiangmen, Zhanjiang, Zhaoqing, Jieyang, and Shaoguan had fewer participants. Twelve participants indicated that they lived in Guangdong but did not specify the city.

### 3.6. Descriptive statistics

Table 1 shows the means and standard deviations of the measurable items and constructs. The calculation results indicate that TS (3.937) and EQ (3.908) scored relatively high. The scores for RI and PA are 3.874 and 3.658, respectively. Generally speaking, when the loadings of each indicator are above 0.7, the reliability of the data is considered acceptable.^[55]^ Table 1 shows that the indicator loadings of the corresponding latent variables range from 0.840 to 0.923, all of which are above 0.7. Therefore, the indicator reliability of each construct is acceptable.

**Table 1:** Mean, standard deviation, factor loadings of measurable items (N = 600).

	Mean	SD	Factor loadings
Experience quality			
EQ1	3.838	0.736	0.840
EQ2	3.944	0.684	0.873
EQ3	3.857	0.709	0.889
EQ4	3.944	0.658	0.889
EQ5	3.955	0.650	0.882
Tourist satisfaction			
TS1	3.982	0.632	0.881
TS2	3.976	0.630	0.904
TS3	3.887	0.669	0.892
TS4	3.904	0.644	0.841
Place attachment			
PA1	3.561	0.817	0.897
PA2	3.712	0.739	0.903
PA3	3.759	0.739	0.892
PA4	3.598	0.787	0.882
Revisit intention			
RI1	3.873	0.665	0.915
RI2	3.878	0.660	0.923
RI3	3.870	0.662	0.919

EQ = experience quality, PA = place attachment, RI = revisit intention, SD = standard deviation, TS = tourist satisfaction.

## 4. Results

The measuring model was assessed by an investigation of the constructs’ internal consistency, employing composite reliability and Cronbach Alpha. The variance inflation factor was utilized to analyze multicollinearity concerns, while convergent validity was determined using the average variance extracted (AVE). Table 2 results indicate that the values of item reliability and the 2 measures of internal consistency are both above 0.70, which is the acceptable threshold proposed by past research.^[45,48]^ Therefore, it can be concluded that the structural EQ, TS, PA, and RI items and internal consistency are satisfactory and reliable. Furthermore, the construct’s AVE exceeds 0.50.^[56]^ Therefore, it is concluded that the structures EQ, TS, PA, and RI have sufficient convergent validity. Finally, the variance inflation factors of the structure range between 2.587 and 2.888, below the recommended critical value of 3.^[57]^ This confirms the absence of problematic multicollinearity in the structural model.

**Table 2:** Constructs reliability and convergent validity.

Construct	Item	Cronbach Alpha	CR	AVE	VIF
Experience quality (EQ)	EQ1 EQ2 EQ3 EQ4 EQ5	0.923	0.942	0.765	2.587
Tourist satisfaction (TS)	TS1 TS2 TS3 TS4	0.902	0.932	0.774	2.888
Place attachment (PA)	PA1 PA2 PA3 PA4	0.916	0.941	0.798	2.835
Revisit intention (RI)	RI1 RI2 RI3	0.908	0.942	0.844	2.840

AVE = average variance extracted, CR = composite reliability, VIF = variance inflation factor.

As shown in Table 3, the square roots of the AVE for all constructs (EQ = 0.875, PA = 0.894, RI = 0.919, TS = 0.880) exceeded the threshold of 0.70, confirming satisfactory convergent validity. According to the Fornell–Larcker criterion, the correlation coefficients between constructs (EQ–PA = 0.658, EQ–RI = 0.772, EQ–TS = 0.854, PA–RI = 0.768, PA–TS = 0.690, RI–TS = 0.799) were significantly lower than the corresponding AVE square roots. This demonstrates adequate discriminant validity of the measurement model, indicating that all latent variables are statistically distinct.

**Table 3:** Discriminant validity (Fornell–Larcker criterion).

Construct	EQ	PA	RI	TS
Experience quality (EQ)	**0.875**			
Place attachment (PA)	0.658	**0.894**		
Revisit intention (RI)	0.772	0.768	**0.919**	
Tourist satisfaction (TS)	0.854	0.690	0.799	**0.880**

Bold values indicate the square roots of the average variance extracted (AVE) for each construct. Discriminant validity is established when the square root of AVE is greater than the inter-construct correlations.

### 4.1. Evaluation of structural model

In the second stage, *I* evaluated the structural model utilizing the path coefficient (beta, explanatory power) *R*², and effect size: *f*².^[46]^ We used bootstrap methods with 5000 resamples to determine the significance of each relational path coefficient.

The results of the structural equation modeling indicated that all hypothesized direct paths were statistically significant (as shown in Table 4). Specifically, EQ had a significant positive effect on PA (β = 0.254, *P* < .001, 95% confidence interval [CI] [0.118–0.382]). EQ also exerted a strong positive influence on TS (β = 0.854, *P* < .001, 95% CI [0.814–0.887]). Additionally, PA significantly predicted RI (β = 0.414, *P* < .001, 95% CI [0.332–0.490]). TS had a significant positive impact on PA (β = 0.473, *P* < .001, 95% CI [0.342–0.610]). Furthermore, TS directly affected RI (β = 0.514, *P* < .001, 95% CI [0.437–0.594]). EQ directly affected RI (β = 0.236, *P* < .001, 95% CI [0.136–0.330]).

**Table 4:** Results of the hypothesized mode.

Hypothesis	Paths	Beta (β)	Standard deviation	*t*-value	*P*-value	95% CI		Decision	*f* ^2^
	Direct effect					LLCI	ULCI		
H1	EQ → PA	0.254	0.067	3.797	.000	0.118	0.382	Supported	0.035
H2	EQ → TS	0.854	0.019	45.184	.000	0.814	0.887	Supported	2.692
H3	EQ → RI	0.236	0.049	4.815	.000	0.136	0.330	Supported	0.057
H4	PA → RI	0.414	0.041	10.150	.000	0.332	0.490	Supported	0.330
H5	TS → PA	0.473	0.069	6.873	.000	0.342	0.610	Supported	0.119
H6	TS → RI	0.514	0.040	12.870	.000	0.437	0.594	Supported	0.508
	Indirect effect								
H7	EQ → PA → RI	0.105	0.032	3.255	.001	0.045	0.172	Supported	
H8	TS → PA → RI	0.196	0.029	6.806	.000	0.140	0.252	Supported	
H9	EQ → TS → PA	0.404	0.060	6.684	.000	0.290	0.526	Supported	
H10	EQ → TS → RI	0.438	0.038	11.532	.000	0.366	0.517	Supported	
H11	EQ → TS → PA → RI	0.167	0.025	6.729	.000	0.119	0.216	Supported	

EQ = experience quality, PA = place attachment, RI = revisit intention, TS = tourist satisfaction.

Effect sizes (*f*²) showed that EQ had a large effect on TS (*f*² = 2.692), while TS had moderate effects on both PA (*f*² = 0.119) and RI (*f*² = 0.508). PA had a moderate effect on RI (*f*² = 0.330), whereas EQ’s effect on PA was small (*f*² = 0.035).

**Figure 2.**
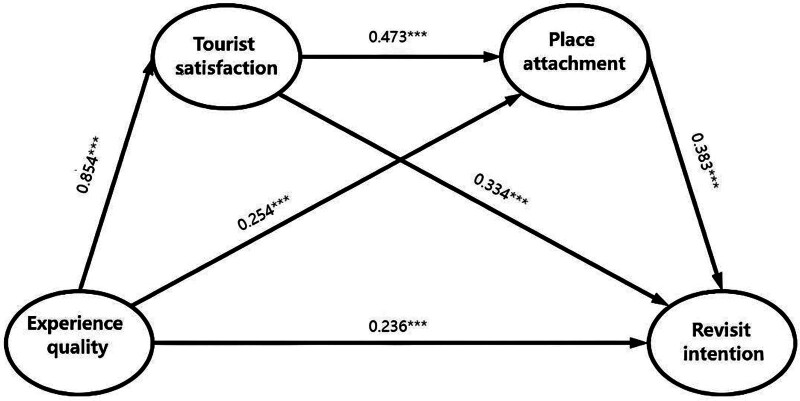
Structural equation modeling. This figure presents the PLS-SEM analysis results with standardized path coefficients. PLS-SEM = partial least squares structural equation modeling.

As the most important criterion, *R*-square predicts endogenous constructs, and its value is higher than 0.25, indicating the strong power.^[58]^ The results indicate that the *R*-squares for TS and PA are 0.729 and 0.493, respectively, while the *R*-square for RI is 0.743. Figure 2 shows the partial least squares structural equation modeling analysis of the proposed model.

### 4.2. Mediation analysis

To assess the level and type of mediation, we utilized the variance accounted for (VAF) test, commonly utilized in contemporary research to demonstrate the mediation of a specific variable.^[45,48]^ These studies demonstrate that a test value < 20% signifies no mediation, a result between 20% and 80% indicates partial mediation, and a result > 80% indicates full mediation. The VAF equals the indirect effect divided by the total effect.

Based on the analysis of mediation effects and VAF, the results indicate that EQ exerts a significant influence on RI through multiple mediating paths, all of which demonstrate partial mediation. Specifically, EQ shows significant effects through the chain mediation of TS and PA (VAF = 41.5%); EQ → PA → RI (VAF = 30.7%), EQ → TS → RI (VAF = 65.1%). In addition, the path from TS to RI via PA (VAF = 27.7%) and the path from EQ to PA via TS (VAF = 61.3%) also exhibit partial mediation.

## 5. Discussion

The study was conducted with the aim of examining the interaction between quality of experience and return intention of outdoor camping tourism as well as the mediator functions of TS and sense of PA under the theory structure of SOR. The findings significantly supported the proposed model and extended knowledge of the mechanisms through which quality experiences determine tourist behavior.

First, the results indicate that EQ significantly enables RI directly and indirectly. Such findings are complemented by previous research, revealing that positive experience evaluations reinforce tourists’ return intention to destinations.^[12,59]^ According to the SOR theoretical conceptualization, EQ is seen as an outside stimulus that elicits internal psychological responses, that is, enjoyment, satisfaction, and attachment, to influence behavioral outcomes. While EQ directly enables RI, its indirect role through TS and PA is stronger, suggesting that emotional and cognitive mechanisms dominate the association from EQ to RI.

Secondly, TS role in mediation was strongly augmented. It was revealed that EQ significantly facilitated TS; thus, it later had a favorable impact toward RI. That is in sync with existing research revealing that satisfaction springs from concurrence of expectations with actual experiences; when expectations are matched or even surpassed, tourists tend to come back.^[60,61]^ In outdoor camping, recreational activity, natural beauty, and environment comfort are paramount, and higher satisfaction is a pivotal predictor of RI.

Third, PA was verified to be a mediator of EQ and RI. Tourists who had undergone excellent camping showed augmented emotional relationships with the destination, hence increasing their RI. This is similar to previous research that finds that PA is not only of functional interdependence but also of symbolic and emotional relationships.^[62,63]^ Contrary to TS, of the immediate kind of judgment, PA is of the deep and long-lasting psychological relationship that can generate lasting loyalty.

Finally, the chain of mediation of TS and PA once more defines the process by which EQ is translated into RI. An exemplary experience triggers first pleasant emotions and satisfaction, then attachment to the destination, and finally RI. The process defines conversion from fleeting emotional reactions to lasting mental connections, substantiating previous research results of emotional–behavioral mechanisms of tourism.^[64,65]^

The research, apart from theory-based outcomes, has practical yields too. Camping experiences need to be enhanced by the management of tourism through advanced facilities, tailor-made services, and actual interactions with nature to cultivate satisfaction and attachment. Policymakers need to comprehend that camping tourism is not merely augmenting the value of the local economy; it is also fostering environmental consciousness and sustainability. Emotionally connected experiences can hence be encouraged through policies that can enhance tourist loyalty and encourage sustainable durability of camping sites.

## 6. Conclusions

This study examines the influence of EQ on RI within the context of outdoor camping tourism, revealing the mediating role of TS and PA. The findings indicate that EQ not only significantly enhances RI but also indirectly strengthens RI through a chain-mediated mechanism between TS and PA. These findings provide strong evidence for the applicability of SOR theory in explaining tourism behavior.

This study makes 3 main contributions. First, at the theoretical level, it clarifies how EQ, as an external stimulus, triggers internal psychological states of satisfaction and attachment among tourists and further drives their RI. Second, at the level of academic literature, this study integrates TS and PA into the chained mediation model, deepening our understanding of the emotional–behavioral conversion mechanism in tourism. Third, at the practical level, this study offers insights for tourism managers and decision-makers: through enhancing service quality, improving facility conditions, and creating emotional experiences, it can effectively strengthen tourist loyalty and promote the sustainable development of camping tourism.

Nevertheless, this study still has some limitations. First, the study employed convenience sampling, which limits the generalizability of the findings. Future research could incorporate more representative and diverse samples. Second, this study used a cross-sectional design; future studies could adopt a longitudinal or experimental design to enhance the reliability of causal inferences.

This study confirms the critical role of high-quality tourism experiences in promoting tourists’ RIs. It not only directly influences behavioral intentions but also has an indirect effect through satisfaction and PA. This study enriches tourism behavior theory by integrating psychological mechanisms with behavioral outcomes, providing practical references for the development of sustainable camping destinations.

## Author contributions

**Conceptualization:** Yanqing Yan, Qiuxian Ye, Xiangfei Zhu, Jifeng Dong, Taiping Li.

**Data curation:** Yanqing Yan, Qiuxian Ye, Xiangfei Zhu, Jifeng Dong, Taiping Li.

**Formal analysis:** Yanqing Yan, Qiuxian Ye, Xiangfei Zhu, Jifeng Dong, Taiping Li.

**Funding acquisition:** Yanqing Yan, Taiping Li.

**Investigation:** Yanqing Yan, Qiuxian Ye, Xiangfei Zhu, Jifeng Dong, Taiping Li.

**Methodology:** Yanqing Yan, Qiuxian Ye, Xiangfei Zhu, Jifeng Dong, Taiping Li.

**Project administration:** Yanqing Yan, Qiuxian Ye, Xiangfei Zhu, Jifeng Dong, Taiping Li.

**Resources:** Yanqing Yan, Qiuxian Ye, Xiangfei Zhu, Jifeng Dong, Taiping Li.

**Software:** Yanqing Yan, Qiuxian Ye, Xiangfei Zhu, Jifeng Dong, Taiping Li.

**Supervision:** Yanqing Yan, Qiuxian Ye, Jifeng Dong, Taiping Li.

**Validation:** Yanqing Yan, Qiuxian Ye, Xiangfei Zhu, Jifeng Dong, Taiping Li.

**Visualization:** Yanqing Yan, Qiuxian Ye, Xiangfei Zhu, Jifeng Dong, Taiping Li.

**Writing – original draft:** Yanqing Yan, Qiuxian Ye, Xiangfei Zhu, Jifeng Dong, Taiping Li.

**Writing – review & editing:** Yanqing Yan, Qiuxian Ye, Xiangfei Zhu, Jifeng Dong, Taiping Li.
